# Advances in the discovery of cathepsin K inhibitors on bone resorption

**DOI:** 10.1080/14756366.2018.1465417

**Published:** 2018-05-03

**Authors:** Jun Lu, Maolin Wang, Ziyue Wang, Zhongqi Fu, Aiping Lu, Ge Zhang

**Affiliations:** a Law Sau Fai Institute for Advancing Translational Medicine in Bone and Joint Diseases (TMBJ), School of Chinese Medicine, Hong Kong Baptist University, Hong Kong SAR, China;; b Institute of Integrated Bioinfomedicine and Translational Science (IBTS), School of Chinese Medicine, Hong Kong Baptist University, Hong Kong SAR, China

**Keywords:** Cathepsin K, osteoclast, bone resorption, osteoporosis, cathepsin K inhibitors

## Abstract

Cathepsin K (Cat K), highly expressed in osteoclasts, is a cysteine protease member of the cathepsin lysosomal protease family and has been of increasing interest as a target of medicinal chemistry efforts for its role in bone matrix degradation. Inhibition of the Cat K enzyme reduces bone resorption and thus, has rendered the enzyme as an attractive target for anti-resorptive osteoporosis therapy. Over the past decades, considerable efforts have been made to design and develop highly potent, excellently selective and orally applicable Cat K inhibitors. These inhibitors are derived from synthetic compounds or natural products, some of which have passed preclinical studies and are presently in clinical trials at different stages of advancement. In this review, we briefly summarised the historic development of Cat K inhibitors and discussed the relationship between structures of inhibitors and active sites in Cat K for the purpose of guiding future development of inhibitors.

## Introduction

Cathepsin K (Cat K), a member of cysteine proteases, is predominantly expressed in osteoclasts and plays crucial roles in degradation of bone matrix composited of hydroxyapatite and protein, especially type I collagen^1^. Both decalcification of hydroxyapatite in an acidic microenvironment and degradation of the protein matrix are inevitable during the bone resorption process. Furthermore, the imbalance between bone resorption (influenced by osteoclasts) and bone formation (influenced by osteoblasts) ultimately results in bone loss, which is significantly associated with osteoporosis^2^^,^^3^. Cat K with a relatively restricted expression pattern exhibits high activity against elastin and type I collagen and is obviously responsible for the relation of osteoclastic bone resorption, which leads to the development of Cat K inhibitors for the treatment of diseases characterised by excessive bone, loss such as osteoporosis^4^^,^^5^.

**Figure 1.**
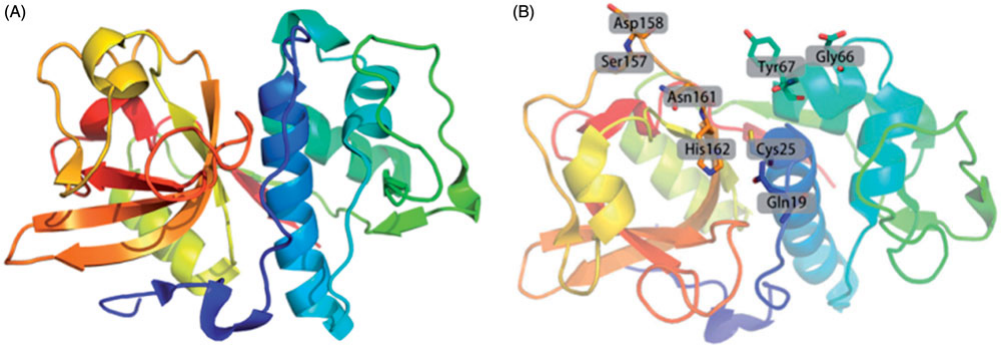
Ribbon drawing of human Cat K and the active sites of Cat K. (A) The overall ribbon structure of human Cat K. The structure is from Protein Data Bank (PDB ID: 5TDI). (B) The residues in active sites of human Cat K.

Moreover, with the clear understanding of the structure of Cat K (Figure 1) and wide consideration of Cat K as a novel target for osteoclast-related maladies, many efforts have been devoted to screening the natural products and developing the therapeutically useful inhibitors for treatment of diseases, such as osteoporosis and other bone disorders displaying excessive levels of resorption^6–9^. At present, research results demonstrated the strong antiresorptive activity and high selectivity of some inhibitors^10^. Furthermore, several Cat K inhibitors under preclinical or clinical investigation for indications such as bone metastases, rheumatoid arthritis or osteoporosis have made optimistic and positive progresses^11–13^.

To date, more than 200 papers on the development of Cat K inhibitors have been published, yielding a large number of compounds with different skeletons, in particular encompassing biologically partial structure of L-leucine. However, only a few reviews on structure–activity relationships of Cat K inhibitors have been published^14^^,^^15^. Considering the importance and attractiveness of Cat K inhibitors in pharmaceutical chemistry and preclinical medicine, we would therefore like to deliver a survey on the recent development in structure–function relationship of Cat K inhibitors.

## Cat K inhibitors

**Figure 2.**
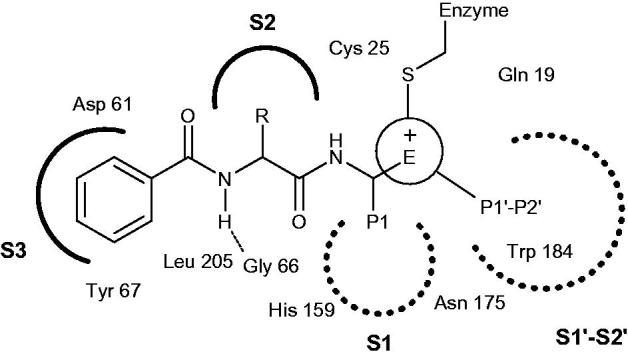
Key binding features of active sites in Cat K.

The search and design of potent and selective active-site Cat K inhibitors for human use have been a highly intense and competitive area over the past 20 years^16–18^. By summing up the experimental results, these inhibitors typically consist of an electrophilic group for covalent binding with active sites in cysteine and some addressed regions for enzyme recognition (Figure 2)^7^^,^^15^. Therefore, according to the available compound sources, the following sections are intended to provide a broad introduction to Cat K inhibitors from literature reports that were generally divided into two classes, synthetic compounds and natural products.

### Cat K inhibitors based on designed synthesis

A large number of competitive efforts have been made, in terms of the disclosure of Cat K as a key role in osteoclast-mediated degradation of bone matrix, in order to design the structures of Cat K inhibitors and evaluate the biological activities and selectivity followed by modulating the chemical moieties for covalent reversible or non-covalent reversible inhibition.

#### Cat K inhibitors based on ketone warhead

**Figure 3.**
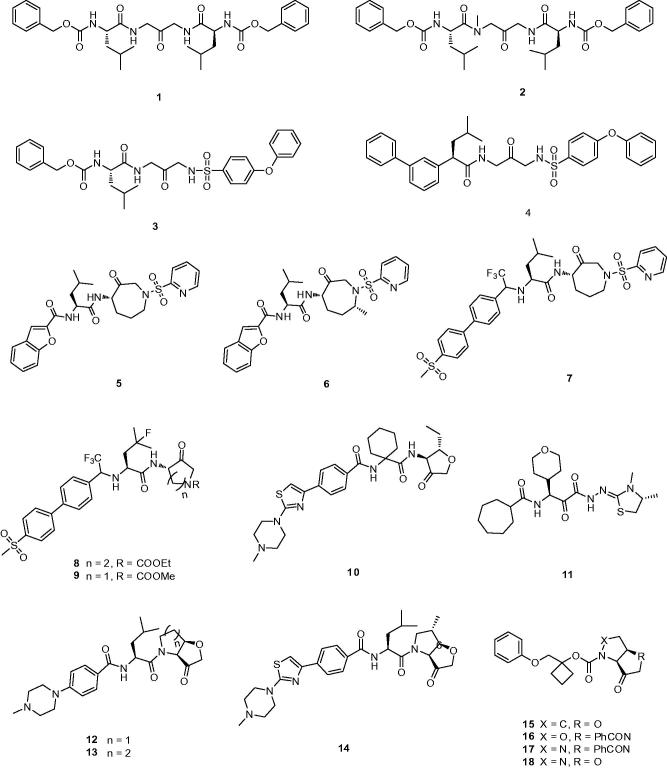
Molecular structures of Cat K inhibitors based on ketone warhead.

In 1997, Veber *et al.* reported a series of selective and reversible Cat K inhibitors based on a poorly electrophilic 1,3-bis(acylamino)-2-propanone scaffold^19^. Through modelling the interaction of active-sites and simplifying the structure of inhibitors, they developed an accessible symmetrical ketone **1** with a *K*_i,app_ value of 22 nM against Cat K (Figure 3). It was interesting to note that **1** exhibited excellent selectivity over other members of cathepsin family (*K*_i,app_ cathepsin L (Cat L), 0.34 μM; cathepsin B (Cat B), 1.3 μM; cathepsin S (Cat S), 0.89 μM)^20^. N-methyl analog of **1** examined effects of methylation, ketone **2**, was 4-fold less active than **1**. Whereas, in order to span the distance of both sides of its active site (picking up the Trp184 aromatic interaction) **3**, the chemical moiety of Cbz-Leu in **1** substituted by 4-phenoxyphenyl sulfonamide, showed 10-fold more active than their original peptide-based lead.

On the other hand, extension of aromatic moiety interacted with Try67, DesJarlais *et al.*^21^ developed a variety of sulfonyl inhibitors, among which **4** with the biphenyl group replacing Cbz showed greater than 500-fold selectivity over Cat B, S, L (*K*_i,app_ Cat K, 1.4 nM; Cat B, >10000 nM; Cat S, 910 nM; Cat L, >1000 nM) (Figure 3). The biphenyl group that best matched the conformation of prime side is more rigid and bulky than the benzyl carbamate. From the analysis of X-ray co-crystal structure, the biphenyl system in **4** occupied the S3 site rather than the substrate backbone binding site and formed an aromatic–aromatic interaction with Tyr67.

Marquis *et al.*^22^ designed an azepanone-basediInhibitor of Cat K **5**, which possessed some special structures including a C-4 chiral center as S and an azepanone ring in a pseudo-boat conformation (Figure 3). The C-4 S stereochemistry was critical for potent inhibition that predicted the higher energy axial orientation bound within the active site of Cat K by molecular modelling. Compound **5**, which incorporated the replacement of the carbonylbenzyloxy group with the benzofuran-2-carboxyamide showed a potently reversible inhibitor of human Cat K with a *K*_i_ = 0.16 nM and a relatively acceptable selectivity against Cat B, S, L (*K*_i,app_ Cat B, 500 nM; Cat S, 4 nM; Cat L, 2.2 nM). Comparison of the transport of cyclic and acyclic analogs, the results from pharmacokinetic analysis revealed inhibitor **5** with cyclic has good oral bioavailability in the rat of 42% with a *T*_1/2_ of 30 min.

**Figure 4.**
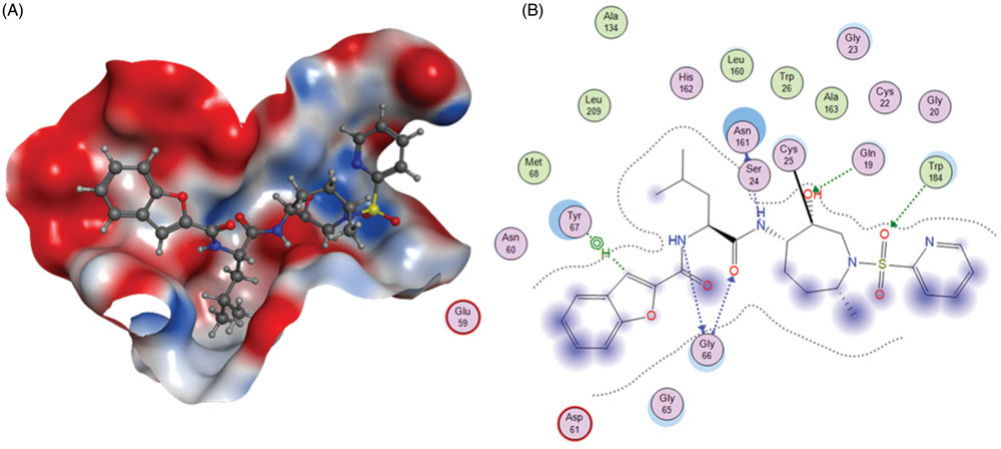
The interactions between **6** and Cat K from molecular modelling. (A) The pocket is shown in electrostatics representation. (B) The detailed interactions between **6** and Cat K. The molecular docking is calculated by AutoDock Vina. Green line: Sidechain hydrogen bond; Blue line: Backbone hydrogen bond; Black line: Covalent bond.

The ketone inhibitors of Cat K pioneered by GSK scientists have been taken a huge number of efforts to realise the desired inhibition and selectivity. The discovery of **6** embodying extremely potent inhibition with picomolar affinity, known as relacatib or SB-462795 (Developed by GSK), was considered as an important milestone (Figure 3)^23^. Compound **6** in a chair conformation has an axial methyl group at C-7 position, which contacts with the S1′ hydrophobic pocket, while the sulfonylpyridine interacts with the S2′ hydrophobic pocket. The interactions between compound **6** and Cat K are shown in Figure 4. Furthermore, conformational analysis revealed that the methyl group at C-4 increased the configurational stability. The 7-methyl substituted azepanone analog shows favorable pharmacokinetic characteristics, good oral bioavailability (89%), and an *in vivo* clearance rate of 19.5 ml/min/kg. However, in spite of those advantages, compound **6** exhibits a rather low or no selectivity over other off-target cathepsins (*K*_i,app_ Cat K, 0.041 nM; Cat L, 0.068 nM; cathepsin V (Cat V), 0.053 nM; Cat B, 15 nM; Cat S, 1.6 nM)^24^.

During the systemic research of odanacatib, Boyd *et al.* investigated that the replacement of nitrile with cyclic ketone warheads was based on the experience of ketones as reversible Cat K inhibitors^8^^,^^25^. Substitution of the benzofuran moiety of compound **5** with an odanacatib-like 4-methylsulfonylphenyl backbone, to provide compound **7**, conceivably allowed interactions on the prime side^26^. The biphenyl of the substrate intensively participates a ring-ring interaction between Tyr67 at the S3 pocket. In spite of this, compound **7** only furnished improved partial selectivity as well as more than 10-fold reduced inhibition.

Merck Frost also provided a series of ketone inhibitors of various cyclic aminoketone ring size and nitrogen substitution, such as **8**, **9** (Figure 3)^27^. The IC_50_ values against humanised rabbit Cat K and selectivity against human Cat L, B, and S exhibited no significant difference compared with inhibitor **7**^28^. Moreover, from the rat bile cannulation study, these inhibitors were rapidly cleared in rats due to cleavage between the amide nitrogen and α-keto stereocenter, as well as oxidation on the ketone heterocyclic.

Medivir UK Ltd. (Little Chesterford, Essex, UK) invented MIV-711, a highly selective Cat K inhibitor; however, the structure of MIV-711 still remains unknown. This company revealed a potent and selective Cat K inhibitor MV061194 (**10**), analog of MIV-711, which featured a reversible ketone electrophile and a piperazine S3 substitute (Figure 3)^29^. With high selectivity, compound **10** was selected as a candidate to be active in reducing bone degradation in preclinical studies (*K*_i,app_ Cat K, 2.5 nM; Cat L, >40,000 nM; cathepsin H (Cat H), >4000 nM; Cat B, >4000 nM; Cat S, >40,000 nM)^30^. Moreover, through degradation of other crucial matrix-embedded growth factors and restricting collagen degradation, compound **10** augmented bone formation probably by preventing Cat K activity.

ONO-5334 (**11**) (Ono Pharmaceutical, Tokyo, Japan) as a potent inhibitor of Cat K with *K*_i_ value of 0.1 nM is undergoing II clinical trials, which showed its inhibitory activity for other cathepsins was 8 to 320-fold lower than that for Cat K (*K*_i,app_ Cat K, 0.1 nM; Cat B, 32 nM; Cat L, 17 nM; Cat S, 0.83 nM) (Figure 3)^12^^,^^31^. Furthermore, **11** has been shown to prevent the decrease in bone mineral density (BMD) in the ovariectomised cynomolgus monkey osteoporosis model and showed a significant increase in BMD compared with placebo and a similar magnitude of suppression on bone resorption compared with the current well known anti-resorptive agents in a 12-month clinical study with postmenopausal osteopenia or osteoporosis^32^.

Quibell *et al.*^33^ stereo-selectively synthesised a series of novel tetrahydrofuro[3,2-b] pyrrol-3-one and hexahydrofuro[3,2-b]pyridine-3-one Cat K inhibitors and demonstrated the *cis*-fused geometry with better inherent stability compared with the corresponding trans-fused, such as **12** and **13** (Figure 3). Although the bicyclic structure was in direct contrast with all other scaffolds based upon substrate-like binding sites, the thiolate addition to **12** was predicted to exhibit the same interaction with P1′–P2′ pocket. The compound **12** is one of the successful applications of *cis*-fused bicycle exhibited as a very potent and selective Cat K inhibitor (*K*_i,app_ Cat K, 8.7 nM; Cat L, >10,000 nM; Cat S, >40,000 nM). Some of preferred compounds similar to **12** have been filed by Amura and presented an improved potency and activity in osteoclast resorption assay^34^^,^^35^. Unfortunately, those compounds showed no or little selectivity against other cathepsin enzymes.

Subsequent patents published a series of several novel nonbasic *cis*-fused 5,5-bicyclic ketone Cat K inhibitors, which possessed a small substituent at the 6-position of the tetrahydrofuro[3,2-b]pyrrol-3-one warhead^36^^,^^37^. By virtue of configurationally stable *cis*-fusion, these compounds were able to access both S1 and S1′–S2′ binding sites. With differently preferred C-6 substituents including methoxyl, amino, heteroatom, hydroxyl or ethyl group, these inhibitors showed considerable affinity^35–37^. Several exemplified compounds exhibited sub-nanomolar inhibition for Cat K, such as **14** (*K*_i app_, 0.6 nM). However, from researching the comparable data, the stereochemistry at C-6 showed no obvious influence on potency.

With the embodied advantages of *cis*-fused bicycle, Amura claimed a series of 5,5-bicyclic ketone Cat K inhibitors, which were based on the replacement of S2 and S3 residues from the aldehyde Cat K inhibitors^38^, for example, **15**, **16**, **17**, and **18** (Figure 3). However, there was no further data provided for describing their inhibition and selectivity.

#### Cat K inhibitors based on nitrile warhead

**Figure 5.**
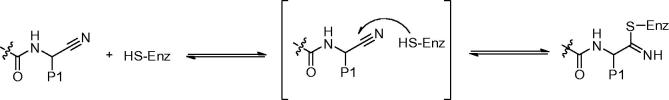
Activity of a nitrile-based Cat K inhibitor is the result of a reversible covalent bond with the active site cysteine of the enzyme.

The electrophilic nitrile warhead has been reported to be an important component for inhibitors of Cat B, K, and S^39–42^. It was well known that the nitrile inhibitors could undergo slow nucleophilic attack of the nitrile moiety to form the reversible thioimidate ester bond between the inhibitor and enzyme (Figure 5). The nitrile warhead as an effect region interacted with active sites of cysteine protease has received much attention during the last decades^43^^,^^44^. The achievement for Cat K inhibitors between potency and selectivity is partly depended on controlling the reactivity of the nitrile warhead.

**Figure 6.**
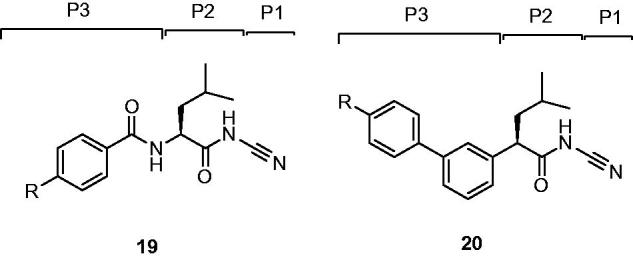
Schematic representation of the assumed binding mode of Cat K inhibitors.

Robichaud *et al.*^46^ replaced P3 amide bond of dipeptide inhibitors **19** with a phenyl ring and gave rise to a series of nonpeptidic biaryl compounds **20** (Figure 6)^45^.

**Figure 7.**
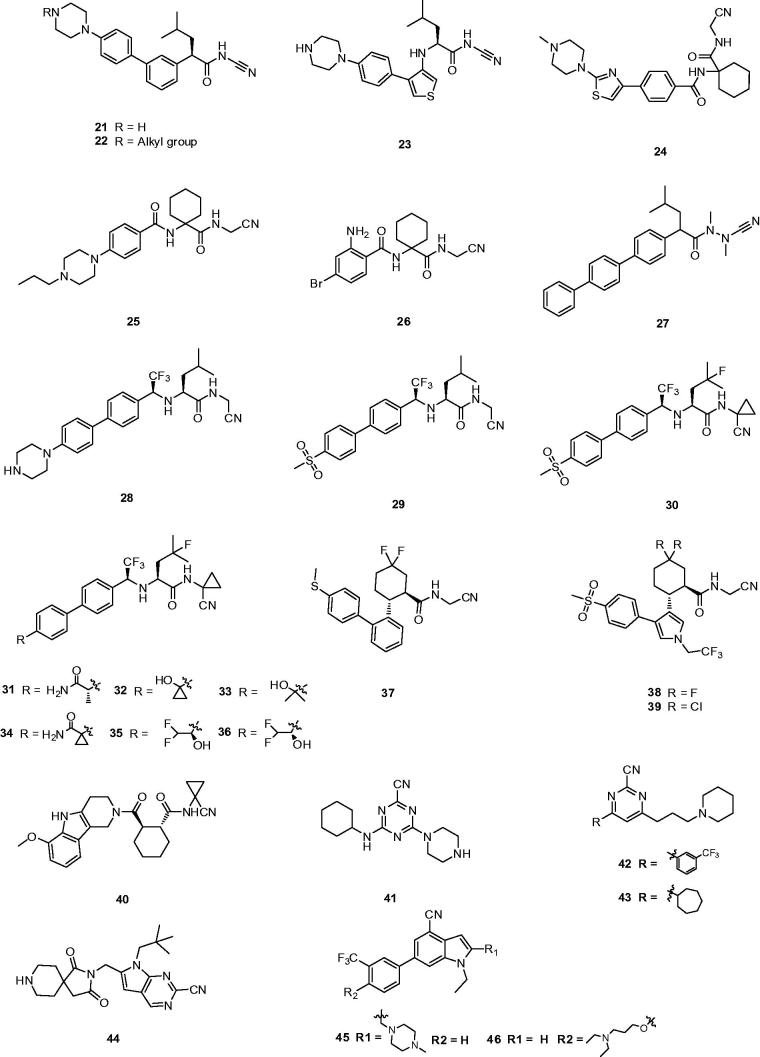
Molecular structures of Cat K inhibitors based on nitrile warhead.

Replaced compound **21** as a potent inhibitor retained activity against Cat K and showed a significantly improved selectivity profile against the other cathepsins (IC_50_ Cat K, 3 nM; Cat B, 3950 nM; Cat L, 3725 nM; Cat S, 2010 nM)^28^. From more experimental data, other alkylated peperazines **22** had similar Cat K potencies but did not show any substantial advantages over the lead unsubstituted piperazine **21** (Figure 7). Moreover, compound **21**, which possessed the inhibition without observably time-dependent, had good pharmacokinetic properties in rhesus monkeys and showed excellent *in vivo* efficacy in the rhesus monkey model for inhibition of bone resorption.

Then, a novel structure of Cat K inhibitor showed that the introduction of a NH linker between P3 aryl and P2 leucinamide moiety formed a H-bond with the Gly66 residue and hopefully enhanced the interaction between inhibitor and Cat K. Preferred compound **23** (Figure 7) showed more than 10-fold improvement in potency over its predecessor **21** and a good selectivity profile against other cathepsins (IC_50_ Cat K, 0.2 nM; Cat B, 123 nM; Cat L, 352 nM; Cat S, 102 nM)^46^. From the model deduction, five-membered heterocycle adequately placed the P3 moiety of **23** into the S3 pocket of Cat K.

Meanwhile, a great deal of efforts from researchers has been made to optimise α-amidoacetonitrile – containing Cat K inhibitors by using a dipeptide template^47^. A series of inhibitors based on a novel tri-ring benzamide moiety and an aminocyclohexanecarboxylate have been provided by Palmer group^48^. For example, the preferred compound **24** (L-006235) (Figure 7), in which 1,1′-cyclohexyl ring occupying P2 position gave excellent binding affinity combined with high selectivity and reduced hydrolysis of the reversible bond, displayed excellent inhibition of cathepsin as well as good selectivity against other cathepsins (IC_50_ Cat K, 0.2 nM; Cat B, 1000 nM; Cat L, 6000 nM; Cat S, 47,000 nM). In addition, the pharmacokinetic study with **24** at 300 mg/kg daily treatment demonstrated that achieved drug exposure was sufficient to inhibit >90% of murine Cat K during the study duration^49^.

Combining previous research experience of introducing a 1,1′-cyclohexyl ring at P2 position and a piperazine moiety at P3 position, researchers from Novartis claimed the N-propylpiperazine substituted compound **25** (Balicatib), also known as AAE-581, in two patents (Figure 7)^50^^,^^51^. Compound **25** as a basic peptidic nitrile inhibitor showed a potent human Cat K inhibition and significantly more selectivity in cell-based assays against other cathepsins (IC_50_ Cat K, 1.4 nM; Cat B, 4800 nM; Cat L, 503 nM; Cat S, 65000 nM)^52^. Balicatib, in a clinical study, showed an increase in bone mineral density and reduction of biochemical markers of bone resorption in the lumbar spine, femur and hips over 18 months of treatment^53^. In phase I clinical trials, balicatib had a well dose-dependent suppression of Cat K and was tolerated with 90% suppression at the 25 mg dosage. In phase II clinical trials, a 50 mg dose of balicatib decreased bone resorption markers serum C-terminal cross-linking telopeptides of type I collagen (61%) and urinary N-terminal cross-linking telopeptides of type I collagen (55%) as normalised to creatineat 1 month^54^. However, compared to *in vitro* enzyme assays, selectivity of balicatib was dramatically decreased in cell-based enzyme assays, due to the lysosomotropic characters which results in basic compounds to accumulate in acidic compartments^52^. Because the Cat B and L are highly expressed in the skin and skin-derived cells, balicatib has been reported to lead to incidences of skin rashes, pruritus, and rare morphea-like skin thickening^55^. With these reverse results, Novartis has claimed to stop the development of this compound.

In order to avoid such side effects caused by basic character, Marjana *et al.* designed a less basic N-(functionalized benzoyl)-homocycloleucyl-glycino-nitrile **26** as a Cat K inhibitor, which revealed high affinity for Cat K with *K*_i_ values and was highly selective for Cat K when compared with Cat L and S (*K*_i,app_ Cat K, 10 nM; Cat L, 11,000 nM; Cat S, >100 μM) (Figure 7)^56^. The kinetic studies showed that compound **26** exhibited reversible tight binding to Cat K, while the X-ray structural studies showed covalent and non-covalent binding between the nitrile group and the Cys25 site.

In the subsequent study, Wu *et al.* reported a new type of Cat K inhibitor **27** with azadipeptide nitrile and without the P2–P3 amide linker, which possessed the favourable balance between potency (*K*_i_ = 0.29 nm) and selectivity of Cat K over other cathepsins (*K*_i,app_ Cat K, 0.29 nM; Cat L, 93 nM; Cat S, 517 nM; Cat B, 2484 nM) (Figure 7)^57^. The study of covalent protein-ligand docking explained the improved selectivity of several representative compounds and this new approach of removing the hydrogen bond donated by the P2–P3 amide linkage of inhibitors to the backbone oxygen of Gly66 in the cathepsin enzymes resulted in highly Cat K-selective aza-nitrile inhibitors.

Black *et al.* disclosed a novel Cat K inhibitor **28** that a trifluoromethyl group replaced the carbonyl of an amide in P2 position and generated a metabolically stable, non-basic amine (Figure 7)^58^^,^^59^. Compound **28** maintained the excellent hydrogen bond to Gly66 instead of an amide. Furthermore, it minimised the basicity of an NH donor to avoid formation of NH_2_^+^ moiety in the biological milieu^60^. This compound was an admirable Cat K inhibitor with IC_50_ < 5 pM and good selectivity over other cathepsin enzymes (IC_50_ Cat B, 1111 nM; Cat L, 47 nM; Cat S, 451 nM). They have arrived at a conclusion that trifluoroethylamine is an excellent surrogate for the P2 amide bond in the inhibitors of Cat K.

**Figure 8.**
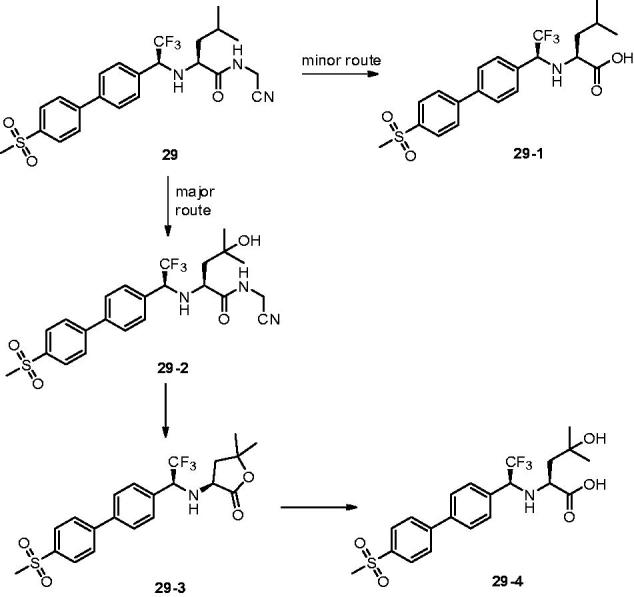
Metabolic pathways for **29** based on *in vitro* and *in vivo* studies.

With the same design concept of keeping trifluoroethylamine amide isostere to enhance potency and selectivity, compound **29** (L-873724) was provided as a potent, selective, and orally bioavailable Cat K inhibitor, which possessed a non-basic P3 substituent (Figure 8)^61^. The methyl sulfone biphenyl analog **29** showed a high potency in the rabbit bone resorption assay and better selectivity than other cathepsins (IC_50_ Cat K, 0.2 nM; Cat B, 5239 nM; Cat L, 1480 nM; Cat S, 265 nM). From the X-ray crystal structure research, the phenyl group in compound **29** adjacent to the CF_3_ group was coplanar with the glycine shelf (Gly66–Gly65) creating a significant hydrophobic interaction. Preliminary pharmacokinetic study revealed a mean decrease of 68% in urinary uNTx/Cr was observed during the six-day on-treatment phase at 3 mg/kg with once-daily oral dosing in the ovariectomised (OVX) rhesus monkey model^62^. The short half-life (2 h) and clearance (Cl =7.5 ml/min/kg) in monkey prevented its further development, while compound **29** has two routes of metabolism.

**Figure 9.**
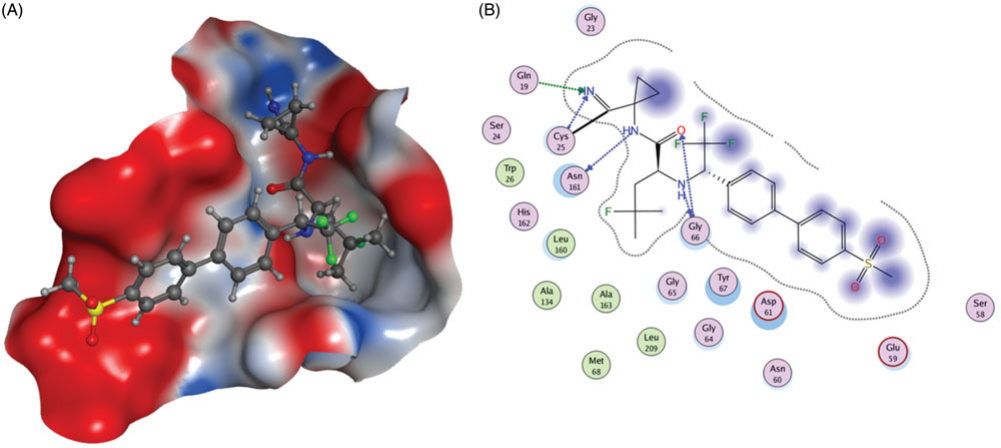
The interactions between **30** and Cat K from molecular modelling. (A) The pocket is shown in electrostatics representation. (B) The detailed interactions between **30** and Cat K. The molecular docking is calculated by AutoDock Vina. Green line: Sidechain hydrogen bond; Blue line: Backbone hydrogen bond; Black line: Covalent bond.

To address the metabolic liability, substituting P1 and modifying P2 moiety of inhibitor L-873724, to give **30** (odanacatib), produced a metabolically robust inhibitor with a long half-life in preclinical species (Figure 7)^63^. The odanacatib directly binds with the side chain of Cys25 of Cat K by forming a covalent bond. The nitrogen atom near the CF_3_ group and the residue Gly66 forms a hydrogen bond. Besides, the Asn161 forms a hydrogen bond with amide group of odanacatib. Furthermore, Gln19 and Gly66 also form hydrogen bonds with odanacatib, as shown in Figure 9^64^. The 4-fluoroleucine side chain at P2 position interacting within the S2 pocket played a crucial role in enhancing the potency and selectivity of odanacatib, which was evaluated in whole cell enzyme occupancy assays (IC_50_ Cat K, 0.2 nM; Cat B, 1034 nM; Cat L, 2995 nM; Cat S, 60 nM)^65^. Compared with balicatib and relacatib, odanacatib still displayed a high potency for decreasing bone resorption markers and increasing BMD in rabbit and monkey ovariectomised osteoporosis models^66^. The randomised placebo-controlled phase I studies showed odanacatib as oral Cat K inhibitor was well tolerated by postmenopausal female, who were treated for 3 weeks (at doses of 5, 25, 50, or 100 mg) or once daily for 21 days (at doses of 0.5, 2.5, or 10 mg) without any significant adverse effects^67^^,^^68^. Interesting, data from phase II indicated women receiving combinations of odanacatib (10–50 mg) for 5 years had gains in spine and hip BMD and showed larger reductions in bone resorption than bone formation markers^69^. Moreover, more than 16,000 patients received odanacatib 50 mg weekly, which increased BMD at the lumbar spine and total hip after 5 years by 11.2 and 9.5% in the phase III trial, respectively, and reduced the risk of hip fracture by 47%, non-vertebral fractures by 23%, and clinical vertebral fractures by 72%^70^^,^^71^. However, odanacatib has been discontinued due to a small increased risk of stroke in the postmenopausal patients, even possessing robust efficacy for treatment of osteoporosis^72^^,^^73^.

Further exploring the strategy that maintained the beneficial features of odanacatib in P1 (cyclopropane) and P2 (fluoroleucine), Isabel *et al.*^74^ disclosed a series of odanacatib analogs **31**–**36** (Figure 7), with various substituted groups in P3 in order to seek novel potent and selective inhibitors. The experimental data *in vitro* or *in vivo* revealed compound **31** with good potency and selectivity suffered from poor oral bioavailability and a complicated metabolic pathway, which led to significant developmental challenges (IC_50_ Cat K, 0.2 nM; Cat B, 288 nM; cathepsin F (Cat F), 718 nM; Cat L, 4266 nM; Cat S, 138 nM). Compound **32** with an alcohol moiety at the benzylic position was lack of selectivity of Cat S (IC_50_ Cat S, 21 nM). Although the dimethyl carbinol analog **33** was highly selective against Cat S with moderate bioavailability in rats of 24%, **33** suffered from a half-life in rats of less than 1 h. Compound **34** failed due to its poor bioavailability in rats (8%) and squirrel monkeys (5%). Interestingly, a pair of enantiomerically pure alcohols, **35** and **36,** were potent and highly selective in *in vitro* assays and exhibited high oral bioavailability as well as long half-lives in rats, rabbits, and rhesus monkeys.

Crane *et al.*^75^ described a series of novel cycloalkylcarboxamides compounds as Cat K inhibitors, in which P2 amide bond was replaced by the cyclohexane moiety, e.g. 37 (Figure 7). The potent enantiomer **37** retained an acceptable selectivity profile against cathepsins L, B, and S (IC_50_ Cat K, 0.28 nM; Cat B, 10080 nM; Cat L, 218 nM; Cat S, 263 nM), and exhibited good potency in the functional bone resorption assay that evaluated the degradation of the type I collagen matrix of bovine bone by isolated rabbit osteoclasts^76^. It appeared that both fluorine substituents in **37** acted cooperatively to produce a synergistic improvement of inhibitor binding to active site of S2 in the Cat K.

Subsequent to the preliminary research, further development found introduction of a methyl sulfone P3-subsitutent and incorporation of five-membered heterocycles as P2–P3 linkers could improve the potency and selectivity of inhibitor, such as **38**, **39**, which were minimally shifted in the bone resorption assay (Figure 7)^77^^,^^78^. Compounds **38** and **39** were quite selective against a wider panel of human lysosomal cysteine proteases including human cathepsins (IC_50_ Cat K, 0.6 nM; Cat B, >9600 nM; Cat L, >2700 nM; Cat S, >2500 nM). Both **38** and **39** displayed acceptable PK profiles in multiple animal species, however, compound **39** exhibited superior bioavailability and half-life.

**Figure 10.**
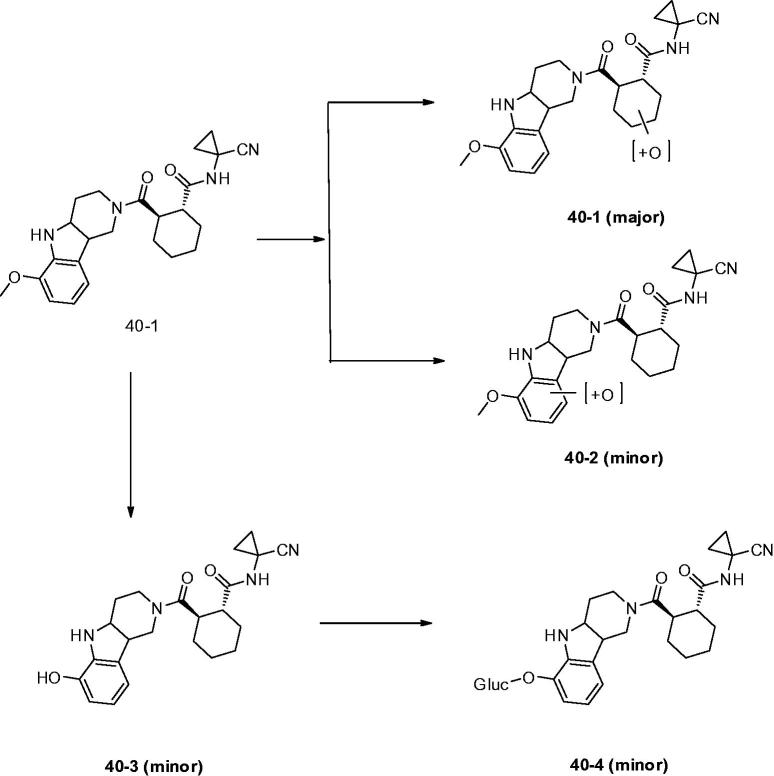
Metabolites found after *in vitro* incubation of **40** with rat hepatocytes.

Dossetter *et al.*^79^ optimised the structures by using small substituents and selected AZD4996 (**40**) as a highly potent and selective Cat K inhibitor (IC_50_ Cat K, <1 nM; Cat B, 2970 nM) (Figure 7). The study of incubation of **40** with rat haptocytes showed a switch of major metabolite to oxidation of the cyclohexyl ring (**40–1**) and reduced oxidation of the carboline aromatic (**40–2**). In addition, a new metabolic route by oxidative demethylation and conjugation was found (**40–3** and **40–4**) compared with other compounds although they were minor metabolites (Figure 10). They revealed key SAR and demonstrated that baseline physical properties and *in vitro* stability by synthesis of compounds with reduced molecular complexity.

Despite the amidoacetonitrile warheads dominate the centre position in the development of Cat K inhibitors, a number of inhibitors based on nitrile heteroaryl chemotypes were still investigated and exhibited excellent potency and selectivity^80^^,^^81^. Novel Cat K inhibitors have been developed based on a 2-Cyano-pyrimidines or triazine. The triazine derivative **41** (Figure 7) as a Cat K inhibitor exhibited high potency against Cat K and selectivity over other cathepsins (IC_50_ Cat K, 1 nM; Cat B, 520 nM; Cat L, 1711 nM; Cat S, 158 nM)^82^. Although **41** showed good stability in human microsomes (*t*_½_ >120 min) and hepatocytes (*t*_½_ = 118 min), it was rapidly cleared in rodent microsomes (rat *t*_½_ = 23 min; mouse *t*_½_ = 4 min) and hepatocytes (rat *t*_½_ = 6 min).

**Figure 11.**
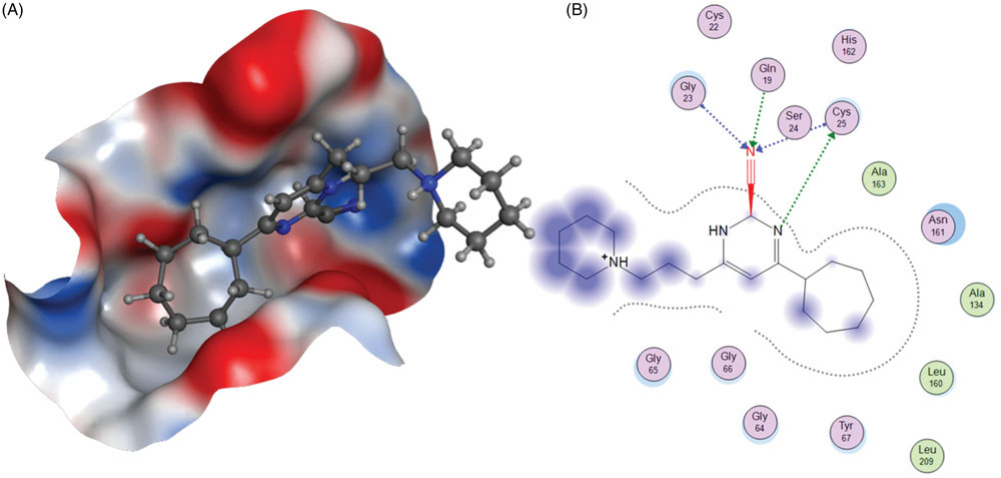
The interactions between **43** and Cat K from molecular modelling. (A) The pocket is shown in electrostatics representation. (B) The detailed interactions between **43** and Cat K. Note: The molecular docking is calculated by AutoDock Vina. Green line: Sidechain hydrogen bond; Blue line: Backbone hydrogen bond.

Rankovic *et al.*^83^ disclosed a novel series of 2-cyano-pyrimidines as potent inhibitors of Cat K. Compound **42** (Figure 7) incorporating a piperidinyl group not only improved Cat K potency (IC_50_ Cat K, 4 nM; Cat B, 4200 nM; Cat L, 10,000 nM; Cat S, 23 nM), but also enhanced solubility (from <1 mg/L to 379 mg/L). Interestingly, 3-CF_3_-phenyl in **42** was replaced with cycloheptyl ring and obtained compound **43**, which showed lower potency but higher selectivity profile against cathepsins (IC_50_ Cat K, 100 nM; Cat B, >10,000 nM; Cat L, 10,000 nM; Cat S, 10,000 nM). From the analysis of X-ray structure, the cycloheptyl ring bound in the S2 pocket, but not as deeply as the 3-CF_3_-phenyl. Moreover, the S2 pocket is slightly narrower in Cat S, which could explain the greater Cat S selectivity displayed by compound **43**, as shown in Figure 11.

Meanwhile, an additional series of Cat K inhibitors have been disclosed in which the central pyrimidine moiety was replaced with an imidazopyridine scaffold^84^. Compound **44** (Figure 7) as a promising Cat K inhibitor exhibited good inhibition activity and selectivity against other cathpsins (IC_50_ Cat K, <1 nM; Cat S, >1000 nM; Cat L, >1000 nM)^85^. It prevented collagen degradation by 73.2% while produced a high concentration in serum. Meanwhile, compound **44** strongly reduced rat CTx with a high concentration even 25 h after administration in the target tissue and showed no signs of toxicity on prolonged administration. In the subsequent patents, extension of C6 and 3-trifluoromethylphenyl substituents has been claimed to provide some dual Cat K/S inhibitors, such as **45** and **46**. It is interestingly observed that moving the basic functionality from imidazopyridine moiety to phenyl moiety can skew the potent selectivity toward Cat S^86^.

#### Cat K inhibitors based on non-covalent interaction

Besides the ketone-based and nitrile-based inhibitors, researchers were also developing non-covalent amide derivatives as potent Cat K inhibitors. Those kinds of compounds do not possess an electrophilic warhead, which could form a covalent reversible bond with the active cysteine residue. However, the lipophilic interactions between aminoethylaniline moiety of the inhibitors and the prime sites in the Cat K furnish to some extent the binding affinity of these non-covalent reversible inhibitors. Meanwhile, the isobutyl or 1,1-cyclothexyl group preferred at P2 and ethyl group preferred at P1 are forming van der Waals with Leu157, Tyr67 etc.^7^^,^^87^.

**Figure 12.**
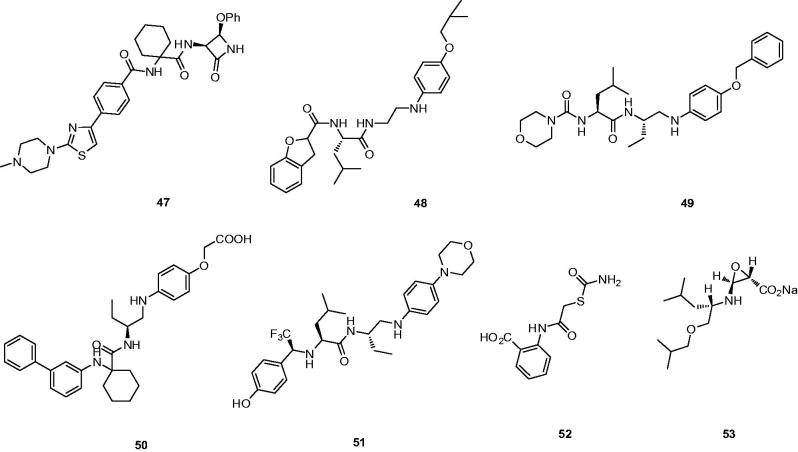
Molecular structures of Cat K inhibitors based on non-covalent interaction.

**Figure 13.**
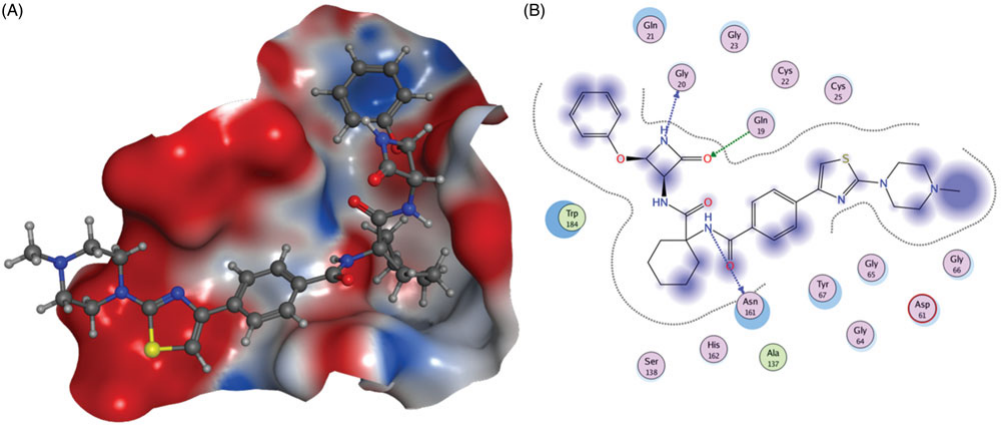
The interactions between **47** and Cat K from molecular modelling. (A) The pocket is shown in electrostatics representation. (B) The detailed interactions between **47** and Cat K. The molecular docking is calculated by AutoDock Vina. Green line: Sidechain hydrogen bond; Blue line: Backbone hydrogen bond.

In order to form a highly favourable ionic bonding interaction with Asp61, Setti *et al.* designed a 3,4-disubstituted azetidinones compound **47** with a basic substituent in the S3 pocket (Figure 12)^88^. Compound **47** exerted its inhibition through direct interaction of Cat K active site with the C-2 carbonyl of the β-lactam, similar to β-lactam reaction with serine proteases^89^. In addition, **47** exhibited excellent potency and selectivity against other cathepsins (IC_50_ Cat K, 4.8 nM; Cat B, 340 nM; Cat L, 2400 nM; Cat S, 17,000 nM), but rat pharmacokinetic results showed **47** possessed high clearance (92 ml/min/kg) and a short MRT (17 min). According to docking result, the Asn161 forms a hydrogen bond with amide group of **47**. In addition, the nitrogen of azetidinone group forms a hydrogen bond with Gly20. The oxygen atom on azetidinone group forms a hydrogen bond with Gln19 (Figure 13).

Altmann *et al.*^90^ reported the discovery of arylaminoethyl amides as novel non-covalent inhibitors of Cat K and probed the structure–activity relationship through incorporation of extended P1 substituents and the replacement of the P3 moiety, exemplifying compound **48** (Figure 12). Compound **48** emerged as the most attractive analog revealed high potency for Cat K inhibition and favourable selectivity profile (IC_50_ Cat K, <3 nM; Cat L,>10,000 nM; Cat S, >10,000 nM)^91^.

Further optimisation of the structure based on the strategy discovered the morpholine derivative **49** (Figure 12), which showed the good Cat K inhibition potency and excellent selectivity over Cat B and L except Cat S (IC_50_ Cat K, 6.8 nM; Cat B, >20,000 nM; Cat L, >20,000 nM; Cat S, 13 nM)^92^.

Sankyo Co., Ltd claimed the 4-aminophenoxyacetic acid **50** as a novel Cat K inhibitor by optimizing the P1, P3, and P1′ units (Figure 12)^93^. The selectivity of inhibitor **50** was achieved as likely to be lack of a covalent interaction with the catalytic cysteine and the potency was maintained on both human and rat (IC_50_ Cat K, 4.8 nM; Cat B,>20,000 nM; Cat L, >20,000 nM; Cat S, 10,000 nM). With biochemical potency on the rat protein and reasonable PK following 50 mg/kg oral dosing (*C*_max_ = 9.2 μg/mL; AUC =103.9 μgh/mL), **50** showed a trend toward increased BMD in OVX rat femur following b.i.d. dosing for 42 days^14^.

Preferred compound **51** (Figure 12) as one of a series of derivatives exemplified was claimed with replacement of trifluorethylamine in the P2–P3 moiety^94^. The non-covalent inhibitor **51** showed the different activity ranges of Cat K inhibition.

With the fast development of computational method for discovery of various inhibitors, Antonio *et al.* identified the first small-molecule allosteric Cat K inhibitor **52** by high-throughput docking of compound libraries to surface sites on the peptidase (Figure 12)^95^. The crystal structure revealed **52** bound to a novel allosteric site on Cat K. Moreover, this compound completely inhibited collagen degradation and had good selectivity for Cat K over related enzymes (IC_50_ Cat K, 80 μM; Cat B, 2300 μM; Cat L, 1600 μM; Cat S, 3300 μM; Cat V, 7700 μM) .

Through screening, Hiroshi *et al.* obtained a potent orally active Cat K inhibitor **53**, which suppressed osteoclastic bone resorption both *in vivo* and *in vitro*^96^. Computer-assisted simulation of the Cat K/**53** complex indicated that **53** blocked the active-site cleft where Cys25 and His162 of Cat K form the catalytic site (Figure 12). Compound **53** with good selectivity is slightly acidic in aqueous solution and thus considered to have less off-target effects than basic inhibitors (IC_50_ Cat K, 34.5 nM; Cat B, 284 nM; Cat L, 582 nM; Cat S, 186 nM).

### Cat K inhibitors based on natural products

As part of a search for novel inhibitors, traditional Chinese medicine has been used as an important source for treatment of various diseases^97^^,^^98^. *Rhizoma Drynariae* (RD) known as “Gu-Sui-Bu” in folk medicine has been frequently utilised in the treatment of bone-related diseases in clinical formulas^99–101^. Some natural products with novel structures from traditional Chinese medicine, microorganisms, or marine organisms exhibited good potency against Cat K^6^^,^^102^.

**Figure 14.**
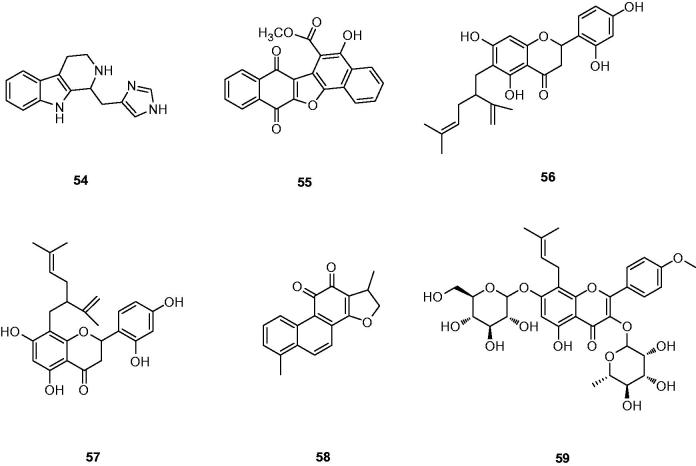
Molecular structures of Cat K inhibitors based on natural products.

Haploscleridamine (**54**) (Figure 14) as a novel tryptamine-derived tetrahydro-β-carboline alkaloid was extracted and characterised by Patil et al.^103^, which displayed the moderate activity against Cat K with an IC_50_ of 26 μM. Unfortunately, no information about selectivity and structureactivity relationship on this inhibitor of Cat K was supplied.

Park *et al.* isolated a furanquinone compound **55** from *Paulownia tomentosa* stem (Figure 14)^103^. Compound **55** showed an IC_50_ value of 21 μM for Cat K. However, it was also found to be capable of inhibiting Cat L, which was closely related to Cat K. Moreover, 5-hydroxyl functional group of **55** could play an important role in the reduction potential of its Cat K inhibitory activity. Although this inhibitory potency of **55** might not be strong enough, it was believed that it still had the potential to be a candidate for designing a new class of Cat K inhibitors.

**Figure 15.**
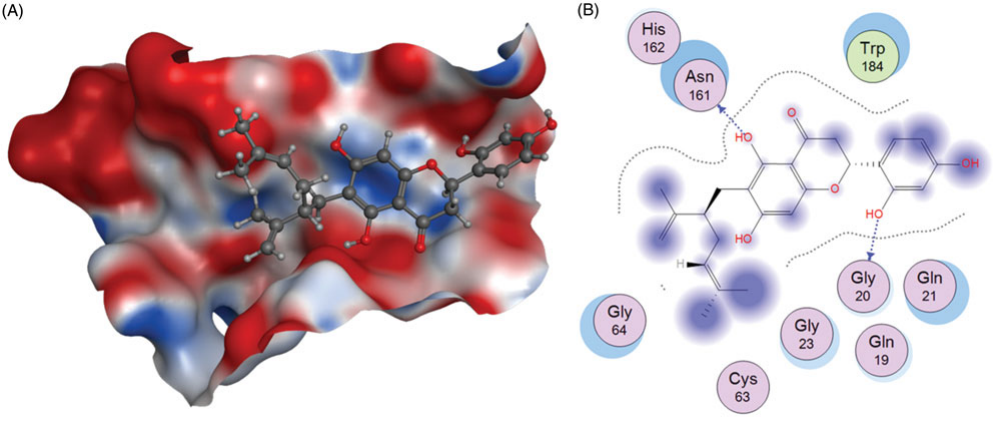
The interactions between **56** and Cat K from molecular modelling. (A) The pocket is shown in electrostatics representation. (B) The detailed interactions between **56** and Cat K. The molecular docking is calculated by AutoDock Vina. Blue line: Backbone hydrogen bond.

Qiu *et al.*^104^ successfully identified Kushennol F(**56**) and Sophoraflavanone G(**57**) to be the active ingredients in *Rhizoma drynariae* that specifically acted on lysosomal enzyme Cat K. Compounds **56** and **57** were shown to suppress Cat K activities with IC_50_ of 8.80 and 27.24 µM, respectively (Figure 14). Using molecular docking and dynamics method, the interactions between these two compounds and protease Cat K were confirmed. As shown in Figure 15, compound **56** formed two hydrogen bonds with Gly20 and Asn161 of Cat K, respectively.

Screening natural product libraries, Brömme *et al.* identified para-dihydrotanshinone (DHT) **58** as a specific collagenase inhibitor of Cat K that did not interfere with the degradation of other biologically relevant substrates (Figure 14)^102^. Meanwhile, **58** revealed a similar outcome of selective inhibition of the collagenase activity of Cat K in osteoclasts when compared with odanacatib (IC_50_ = 6.2 µM)^105^. The study of molecular docking and binding experiments suggested that **58** bound to a specific exosite in Cat K, which was crucial for the formation of collagenolytically active oligomers in the presence of GAGs^106^.

Icariin (**59**), the major pharmacologically active flavonol diglycoside contained in the Epimedium family, had bone-strengthening properties and has been used to promote healing of bone fractures or prevent osteoporosis (Figure 14)^107^. Moreover, Hsieh *et al.*^108^ reported **59** had the inhibition of osteoclast differentiation and bone resorption. Pang *et al.* revealed **59** with the maximum concentration tested 1 mM had 64% inhibition of Cat K activity^109^. Unfortunately, there was no further report about the selectivity compared with other cathepsins.

## Conclusions

Over the past decades, especially the confirmation of tight relationship between Cat K and bone-related diseases like osteoporosis, a large number of efforts have been devoted on developing the Cat K inhibitors. The nitrile and ketone warheads particularly played the prominent roles. Cat K possesses a series of active residues, among which catalytic cysteine Cys25 forms an irreversible or reversible covalent bond with different kinds of inhibitors through a hydrazide, vinylsulfone, epoxide, aldehyde, ketone, or nitrile. Irreversible covalent inhibitors at early stage used in preclinical study led to antigenic and immunologic complications characterised by the results of pcynodysostosis in humans and osteopetrosis in mouse model^8^^,^^110^^,^^111^. Subsequently, reversible covalent or non-covalent inhibitors started another chapter for the research of Cat K. Introducing basic groups or modulating basic functionality in the S3 pocket could compensate for decreased affinity of the non-covalent binder for Cat K. It was noted that the basic nitrogen provided the potential to interact with Asp61 in the S3 pocket of Cat K resulting in increased potency and selectivity^112^. However, the inhibitors containing excessively basic functionality could reach concentrations in acidic lysosome and be sufficient to cause other off-target effects despite excellent biochemical selectivity. It is essential to control selectivity by making use of of non-covalent interactions for the initial molecular recognition. In addition, creating the suitable chemical moiety renders other interaction, like van der waals between P2 substituent and Leu157, Tyr67 in S2 pocket. Moreover, excellent binding affinity could be achieved by making use of the reversible binding nitrile and ketone warheads. In the view of overall potency, the synthesised compounds have obvious advantages against the Cat K in the selectivity and inhibition. However, some compounds in the preclinical or clinical trials were still facing with the challenges in unavoidable side effects and limitations. Some strategies of altering the delivery systems were demonstrated to improve the bioavailability and reduce side effects by forming the conjugate via encapsulation or suitable linkers^113–116^. In a word, a large number of synthesised compounds and characterised natural products offered a relative clear structure–activity relationship for the future design and development of Cat K inhibitors.
